# Clever strategists: Australian Magpies vary mobbing strategies, not intensity, relative to different species of predator

**DOI:** 10.7717/peerj.56

**Published:** 2013-03-19

**Authors:** A Koboroff, G Kaplan, LJ Rogers

**Affiliations:** Centre for Neuroscience and Animal Behaviour, University of New England, Australia

**Keywords:** Risk assessment, Mobbing, Anti-predator behaviour, Australian Magpie

## Abstract

Anti-predator behaviour of magpies was investigated, using five species of model predators, at times of raising offspring. We predicted differences in mobbing strategies for each predator presented and also that raising juveniles would affect intensity of the mobbing event. Fourteen permanent resident family groups were tested using 5 different types of predator (avian and reptilian) known to be of varying degrees of risk to magpies and common in their habitat. In all, 210 trials were conducted (across three different stages of juvenile development). We found that the stage of juvenile development did not alter mobbing behaviour significantly, but predator type did. Aerial strategies (such as swooping) were elicited by taxidermic models of raptors, whereas a taxidermic model of a monitor lizard was approached on the ground and a model snake was rarely approached. Swooping patterns also changed according to which of the three raptors was presented. Our results show that, in contrast to findings in other species, magpies vary mobbing strategy depending on the predator rather than varying mobbing intensity.

## Introduction

For effective defence against predation, potential prey must be able to accurately and rapidly assess risks and react appropriately. The level of intensity of any anti-predator responses may be associated with assessment of a number of factors. Such factors include the proximity of the predator to the potential prey (Creswell, 1993; Albrecht & Klvaňa, 2004; Sordahl, 2004; Kleindorfer, Fessl & Hoi, 2005), the species of predator (Edelaar & Wright, 2006), posture of the predator (Hamerstrom, 1957; Coss & Ramakrishnan, 2000), previous experience (Kruuk, 1976) and behaviour of the predator (Edelaar & Wright, 2006; Griesser, 2008).

In a previous study, we found that Australian Magpies (*Gymnorhina tibicen*) produce mobbing calls that are specific to predator species (Kaplan et al., 2009). In response to three taxidermic models (two species of eagle and a Lace Monitor Lizard, *Varanus varius*), magpies produced a range of vocalisations but one particular vocalisation was elicited only by eagles. Interestingly, the number of vocalisations elicited by the different predator species did not vary. Hence we concluded that magpies discriminate between predators although they do not vary their calling intensity according to type of predator. This is perhaps unusual considering that mobbing intensity is usually described as the number of mobbing calls given (e.g. Owings & Loughry, 1985; Graw & Manser, 2007). We therefore investigated whether non-vocal, anti-predator behaviour of magpies varied according to predator species.

Nest defence theory (Knight & Temple, 1986; Montgomerie & Weatherhead, 1988) predicts that mobbing intensity increases with the age of the nestlings because, for example and amongst a number of other reasons (see Montgomerie & Weatherhead, 1988), the offspring become more conspicuous to predators (Redondo, 1989) or re-nesting potential decreases as the breeding season progresses (Curio, Regelmann & Zimmerman, 1984). We also tested whether the stage of juvenile development (i.e. after fledging) might influence the anti-predator response. For example, Californian Ground Squirrels (*Spermophilus beecheyi*) decrease their intensity of mobbing of Pacific Rattlesnakes (*Crotalus oreganus*) as their pups age (Swaisgood, Owings & Rowe, 1999; Swaisgood, Rowe & Owings, 2003). We thus predicted similar strategies might apply in magpies; that is, mobbing intensity would decrease as age of the juveniles increased. Hence, we presented groups of magpies with five species of predators across three distinct stages of juvenile development (developmental stage is defined below).

It was also possible that mobbing intensity would vary according to the importance of birds in the predators’ diet. Based on this assessment, we predicted that the Little Eagle (*Hieraetus morphnoides*) would represent the greatest threat since up to 77% of its diet may consist of birds (Debus, 1984). The Brown Goshawk (*Accipter fasciatus*) would be next since this species is also an avid bird hunter and birds constitute 37–66% of its total diet (Marchant & Higgins, 1993). The Wedge-tailed Eagle (*Aquila audax*) feeds primarily on mammals, birds representing only 10–28% of its diet (Leopold & Wolfe, 1970; Brooker & Ridpath, 1980; Baker-Gabb, 1984; Sharp et al., 2002) and may, perhaps, be a lesser threat to magpies than any of the other raptors.

Reptilian predators may also elicit mobbing behaviour. The diet of Lace Monitor Lizards is 18% birds and stomach content analysis has found evidence that birds as large as Currawongs (*Strepera graculina*), a species closely related to magpies, are consumed (Weaver, 1989). We also presented a model of a snake, which closely resembled two species: Brown Snake (*Demansia textilis*) or a Copperhead (*Austrelaps superbus*). Neither of these species is known to prey on birds that have fledged (Shine, 1987; Shine, 1989). In a previous study, however, we presented the same model snake to other groups of magpies and they did show some anti-predator behaviour such as alarm calling (Koboroff & Kaplan, 2006).

The five predator species used as models vary considerably in size and hunting techniques. Monitor Lizards seek out prey primarily by scent and, by climbing trees (King & Green, 1999), whereas snakes are primarily ambush predators and, like the lizard, may climb trees or attack on the ground. Wedge-tailed Eagles, the largest of the Australian raptors, use glide attacks, direct-flying attacks, or tail-chasing with the majority of prey being captured on the ground (Marchant & Higgins, 1993), whereas Little Eagles, which are substantially smaller than the Wedge-tailed Eagle and very agile, attack in flight from high altitudes or from perches (Debus, 1984). By contrast, the Brown Goshawk is mainly an ambush hunter and it flushes out prey, the majority of its attacks relying on stealth and surprise (Marchant & Higgins, 1993). There is evidence that nestling, juvenile, and even adult magpies are occasionally taken by each of these predators apart from the snake (Bravery, 1970; Leopold & Wolfe, 1970; Brooker & Ridpath, 1980; Debus, 1984). All of the selected species were native to the area in which the experiment was conducted.

## Materials and Methods

### Animals and study site

Prior to any breeding attempts, magpies typically form flocks. Once a male and female pair-bond, they seek a territory but may not always succeed and remain semi-nomadic or marginal to a group that occupies a permanent territory (Carrick, 1972). Only the most successful pairs manage to form and maintain permanent territories and breed successfully (Carrick, 1972; Farabaugh, Brown & Hughes, 1992; Kaplan, 2008). Hence, our study involved only groups that occupied permanent territories and produced offspring. We did not need to band the magpies because their wing markings are individually distinct and easily identifiable and we took photographs of these wing markings for reference in later trials. We selected 14 groups with territories that were easily accessible and observable, each group consisting of several magpies (6.2 ± 0.6, mean and standard error, magpies per group). A total of 87 magpies were part of the study. All groups were located within the city limits of Armidale, New South Wales, Australia (30°32′ S, 151°40′ E). The habitat is best described as open woodland with a combination of native *Eucalypt* species and exotic vegetation.

### Model predators

**Figure 1:**
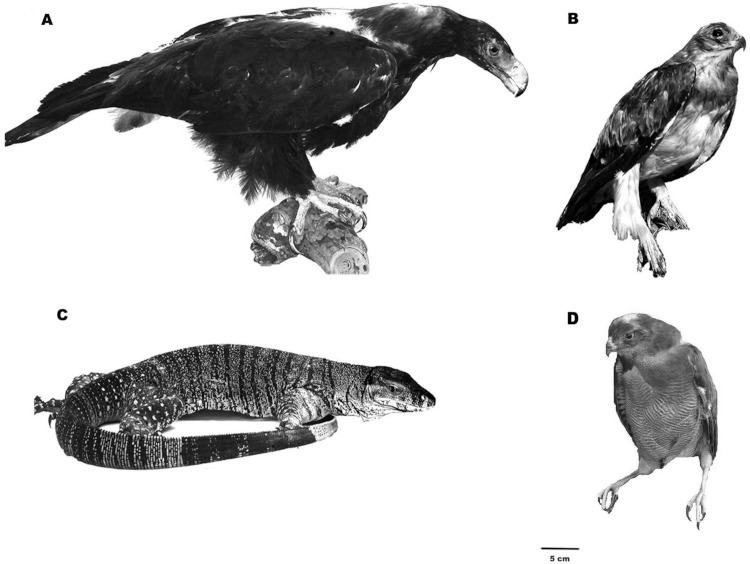
Images of the taxidermic models presented. Taxidermic specimens presented to the groups of magpies. A, Wedge-tailed Eagle; B, Little Eagle; C, Lace Monitor Lizard; D, Brown Goshawk. The images are scaled to size, 5 cm bar indicated. This figure has been modified from Figure 1 in Kaplan et al. (2009).

The three birds of prey (Little Eagle, Wedge-tailed Eagle and Brown Goshawk) and the Lace Monitor Lizard were taxidermic specimens (Fig. 1). The model snake was made from rubber (130 cm in length and 5.0 cm in circumference at widest point). The snake was dull in colour and resembled two locally extant snake species.

### Testing procedure

This study was conducted over a 12-month period from September 2005 to August 2006. Testing took place between 07:00 and 11:00 h (Australian Eastern Standard Time). The model predator was covered by a cloth and placed on the ground in or near the centre of the group’s territory. The models were stationary and placed on the ground approximately in or near the centre of the group’s territory. The experimenter revealed the model by slowly removing the cover, ensuring that the feathers were not disturbed and the model remained in position. He then walked slowly away to at least 20 m from the stimulus and hid behind a nearby structure or tree.

We recorded the magpies’ behaviour for 5 min immediately following their detection of the model predator. Detection was considered to have occurred when at least one magpie approached to within 5 m of the stimulus or emitted an alarm vocalisation, be this a generic or specific eagle alarm call as described elsewhere (Kaplan et al., 2009). A non-response to the stimulus was recorded when, after 30 min, the magpies did not alarm vocalise or approach.

The behaviour of the magpies was videotaped using a Panasonic digital video recorder (NVGS35), with the video frame centred on the model predator and a field of view of at least a 12 m diameter. In addition, observations in the field were noted using pen and paper.

Using all five predator models, the groups were tested regularly, covering three stages of juvenile development post-fledging (210 trials). Stage 1: September to December, the juveniles had recently fledged (at this time, offspring are at their most vulnerable) and, for up to 3 months, are still being fed by the adults (Kaplan, 2008). Stage 2: February to April, juveniles aged approximately 5–7 months feed independently and move about by themselves or in sibling groups. Stage 3: June to August, juvenile birds begin to disperse (8 to 10 months of age; Kaplan, 2008). Hence, the three stages of testing signified varying degrees of juvenile dependence on the adults. Each predator model was presented once per group per stage of development (70 trials per stage). The order of presentation of the models was randomised within a testing stage. The interval between trials was at least 48 h per group.

### Behaviour scored

Behaviour of each group of magpies was scored from the video footage. The entire group was scored using a continuous sampling technique (Martin & Bateson, 1993), scored per group and as bouts. The following behaviour was scored:

(1) Swooping: a flight towards and coming within 1 m of the stimulus, usually approaching the predator from behind. The number of swoops scored included all swooping flights that came within 1 m of the stimulus. Two types of swooping flight patterns were observed: (1a) a direct flight towards the model predator, swooping at it and continuing in the same direction (direct swoop), (1b) a direct flight towards and swooping of the model predator but, instead of continuing in the same direction, the magpie turned in mid air and returned to swoop at the model again in a steep downward flight (looping swoops). Looping and direct swoops were scored separately.

(2) Beak claps: performed while on the ground and moving quickly towards the model predator with extension of the magpie’s neck so that the beak (generally opened) came close to touching the model and the beak was clapped shut while near the head of the model predator.

(3) Jumping: leaping up (approximately 0.5–1.5 m above the ground) with one or two flaps of the wings and landing on the ground again without taking flight during the jumping event.

(4) Moving around the stimulus (circling) while on the ground at less than 3 m distance from the stimulus.

(5) Stationary viewing: the bird was stationary on the ground within 5 m of the model predator and fixating it using one or the other of its lateral fields of vision, often turning the head from side to side for several monocular fixations.

The number of magpies present (scored as the number of individuals that approached the model predator within a 5 m radius) and the number of times the birds made physical contact with the models were also scored.

### Statistical analysis

Since the number of magpies per group varied, all scores were standardized by dividing the total number of events by the number of magpies present. Using the standardised scores, we used non-parametric tests because our data did not meet the assumptions of parametric tests even after several transformations were attempted. Hence, Friedman’s tests were conducted with type of predator as the repeated measure and with each data point representing one trial from one group.

All data were analysed using SPSS version 12.0.0 (2003). Bonferroni adjustments were made to account for Type II errors of multiple comparisons.

The project had Animal Ethics Approval by the University of New England (AEC05/095, AEC 06/092) and was approved and conducted under license (S10361), by the National Parks and Wildlife Division of New South Wales, Australia.

## Results and Discussion

### General observations

The magpies typically detected the model predator within the first minute of it being revealed. The majority of responses to four of the five model predators (Little Eagle, Wedge-tailed Eagle, Brown Goshawk and Monitor Lizard) were indicative of mobbing (i.e. loud and repetitive alarm vocalizations and congregation of the group around the stimulus) but the suite of behaviour and style of the approach varied depending on the species. By contrast, no mobbing or avoidance behaviour was elicited by the snake model; the magpies rarely approached it (2 out of 42 trials) and, in the two cases of approach, it was only briefly (<1 min) and the birds performed only one or two bouts of stationary viewing. Therefore, responses to the snake model were not analysed.

We also checked to see whether mobbing responses might vary in any way consistent with group size. To do this we correlated each of the measured mobbing responses (standardised scores) with group size and found no significant relationships (Spearman’s rho ranged from −0.41 to 0.49 and *P* values from 0.08 to 0.95). Variance of these measures also had no significant relationship with group size (*r* values ranged from −0.38 to 0.52 and *P* values from 0.06 to 0.97), meaning that the magpies were not more audacious in larger groups and all adults in a group participated.

### Effect of stage of juvenile development on anti-predator behaviour

**Figure 2:**
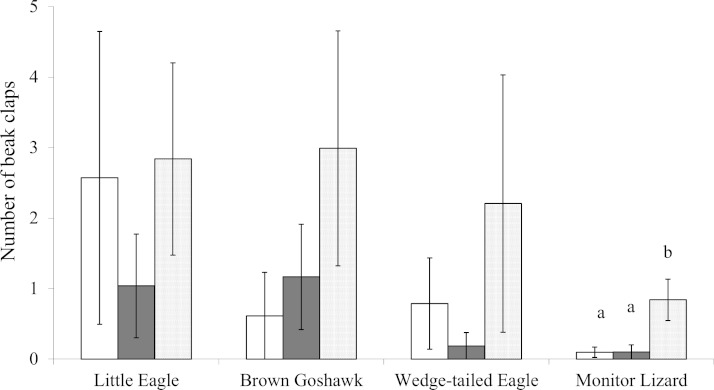
Beak clapping. Beak clapping behaviour elicited by the model predators presented at each stage of juvenile development. White bars indicate Stage 1 of juvenile development, gray bars indicate Stage 2 and the dotted bars indicate Stage 3 (see text for details). Bars marked *a* differ significantly from those marked *b*. Means and standard errors are presented.

There was no significant effect of stage of juvenile development on swooping, jumping, circling or stationary viewing regardless of which one of the predators was presented (${\chi }_{13}^{2}$ ranged from 0.0 and 5.2, and *P* values ranged from 0.53 to 1.00). The number of beak claps elicited by the Monitor Lizard, however, varied significantly with stage of juvenile development (Fig. 2; ${\chi }_{13}^{2}=9.9$, *P* = 0.01): the magpies performed more events of beak clapping during Stage 3 of development than during Stages 1 or 2 (Fig. 2; Stage 1 vs Stage 3, *Z* = −2.0, *P* = 0.04; Stage 2 vs Stage 3, *Z* = −2.4, *P* = 0.018). The number of beak claps elicited by the other model predators did not vary significantly with stage of development of the offspring (Little Eagle: ${\chi }_{13}^{2}=1.0,P=0.64$; Wedge-tailed Eagle: ${\chi }_{13}^{2}=1.3,P=0.53$; Brown Goshawk: ${\chi }_{13}^{2}=3.1,P=0.21$).

According to the findings by Swaisgood and colleagues (2003), the most intense responses to the predators should occur during Stage 1 and the least intense during Stage 3. Our data show, however, that the anti-predator behaviour of Australian Magpies does not vary greatly over the three stages of juvenile development tested. The exception was beak clapping at the Monitor lizard, which increased significantly during Stage 3. Hence, our findings are in contrast to those of Swaisgood and colleagues (2003). However, it is possible that the increased intensity of beak clapping only during Stage 3 resulted from nest building by the adult birds for a subsequent generation. Monitor Lizards are known nest predators (Weaver, 1989; King & Green, 1999; and personal observations) and are therefore a threat to breeding magpies. Therefore, although the juveniles at Stage 3 of development are close to maturity and about to disperse from their natal territories (Kaplan, 2008), the increased beak clapping may have reflected nesting behaviour rather than being a reference to protection of existing juveniles and this may explain our findings. Nevertheless, it was unexpected (see Introduction) that stage of juvenile development would have no major effect on the mobbing responses.

### Effect of predator type on anti-predator behaviour

**Figure 3:**
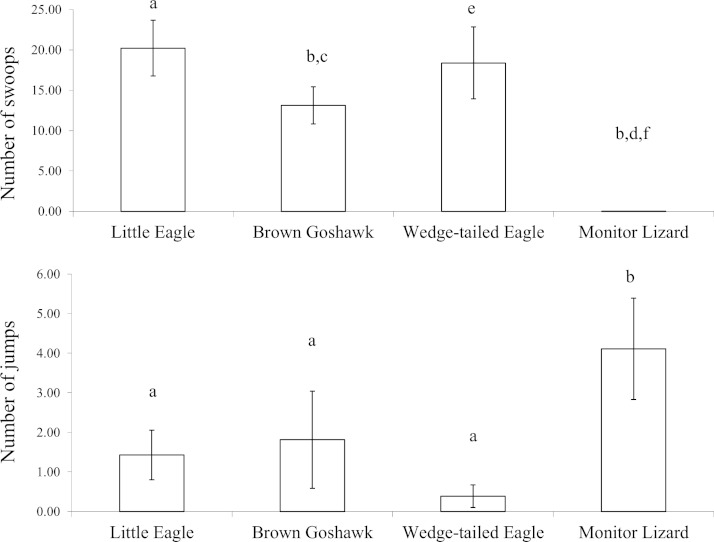
Agonistic behaviour. Agonistic behaviour elicited by the model predators across all stages of development: Top, swooping (all types) and bottom, jumping. Bars marked *a* are significantly different from those marked *b*, *c* from *d* and *e* from *f*. Means and standard errors are presented. The data for the snake were excluded from analysis due to failure of the magpies to mob this stimulus.

Since there was no effect of the stage of juvenile development on anti-predator behaviour elicited by the various model predators (with the exception of the number of beak claps elicited by the Monitor Lizard), the data were collapsed across the three stages of development. The number of swoops (direct plus looping swoops) varied significantly according to predator type (${\chi }_{3}^{2}=24.8,P=0.00$ [Bonferroni adjusted: α = 0.0125]) (Fig. 3). All of the raptor models elicited significantly more swoops (direct plus looping swoops) than did the Monitor Lizard (*Z*-values ranged from −3.2 to −3.3; *P* = 0.00). Indeed, only three swoops were scored in total over all presentations of the Monitor Lizard. Significantly more swoops were scored during presentations of the Little Eagle than during presentations of the Brown Goshawk (*Z* = −2.0, *P* = 0.05). Comparisons of the number of swoops elicited by the Little Eagle and the Wedge-tailed Eagle (*Z* = −0.5, *P* = 0.63), as well as by the Brown Goshawk and the Wedge-tailed Eagle (*Z* = −1.5, *P* = 0.20), revealed no significant differences.

The scores of jumping showed a pattern opposite to that of swooping (Fig. 3). The number of jumps varied significantly according to type of predator (${\chi }_{3}^{2}=24.5,P=0.00$ [Bonferroni adjusted: α = 0.0125]). Higher numbers of jumps were elicited by the lizard than by the raptor models (*Z* values ranged from −2.3 to −3.1; *P* values ranged from 0.00 to 0.02). The number of jumps did not vary significantly according to which raptor was presented (*Z* values ranged from −0.5 to −1.8; *P* values ranged from 0.07 to 0.60).

The magpies made contact with the raptors (either with the beak or feet). This varied depending on the raptor species presented. By contrast, the magpies did not make contact with the lizard or the snake. Considering all scores of contact of the model regardless of the stage of juvenile development, the Goshawk model alone scored 54% (126 events) of all contact events. The Little Eagle and Wedge-tailed Eagle elicited considerably fewer contact events (32% or 74 events, and 14% or 33 events respectively).

The magpies performed a statistically similar number of beak claps during all presentations regardless of which predator species was presented (Stage 1: ${\chi }_{3}^{2}=1.9,P=0.59$; Stage 2: ${\chi }_{3}^{2}=5.2,P=0.16$; Stage 3: ${\chi }_{3}^{2}=0.7,P=0.86$).

**Figure 4:**
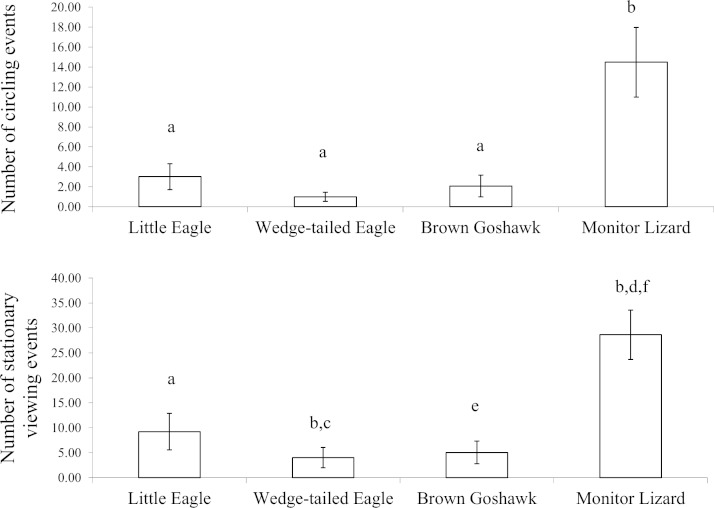
Inspection behaviour. Inspection behaviour elicited by the model predators across all stages of development: (Top) circling and (Bottom) stationary viewing. *a* is significantly different from *b*, *c* from *d* and *d* from *e* and *e* is significant different from *f*.

Circling and stationary viewing occurred primarily during presentations of the Monitor Lizard (Fig. 4). The latter elicited 70% of all circling events and 61% of all stationary viewing events and there was a significant main effect of predator species for these two measures (Circling: ${\chi }_{3}^{2}=14.11,P=0.00$; Stationary Viewing: ${\chi }_{3}^{2}=18.6,P=0.00$). There were significantly more circling events (*Z* values ranged from −2.6 to −3.1; *P* = 0.00) and stationary viewing events (*Z* values ranged from −2.5 to −3.1; *P* values ranged from 0.00 to 0.01) in response to the Monitor Lizard compared to the model raptors. There was no significant difference in the number of circling events elicited by the different species of raptors (*Z* values ranged from −0.7 to −1.1; *P* values ranged from 0.26 to 0.48). However, the Little Eagle elicited more stationary viewing than did the Brown Goshawk (*Z* = −2.0, *P* = 0.04).

**Figure 5:**
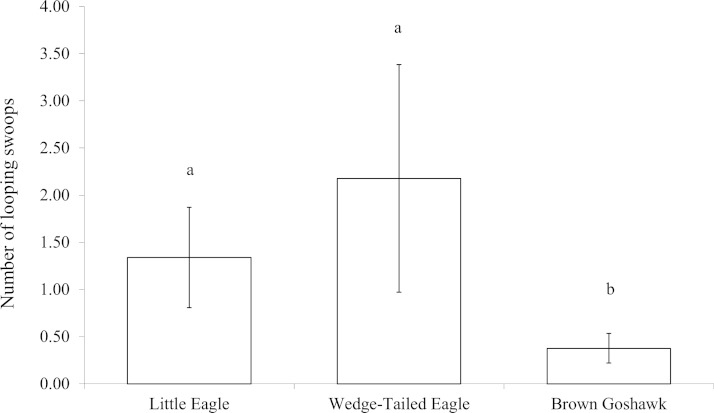
Looping swoops. Number of looping swoop flights performed when mobbing raptors. Means and standard errors are presented. Bar marked *b* differs significantly from those marked *a*.

The scores for swooping included looping and direct swoops. Considering the looping swoops separately, because they potentially exposed the magpie to increased risk of predation, there was a significant difference in the number of looping swoops made according to predator species (${\chi }_{13}^{2}=6.720,P=0.035$). As Fig. 5 shows, the Brown Goshawk elicited significantly fewer looping swoops than did the Little Eagle (*Z* = −2.0, *P* = 0.05) and the Wedge-tailed Eagle (*Z* = −2.2, *P* = 0.03). Scores of direct swooping did not vary significantly with stimulus type (*Z* = 0.452, *P* = 0.798).

Since habituation to stationary model predators has been found in other species (Shalter, 1978; de Azevedo, Young & Rodrigues, 2012), we checked whether this might have occurred in our sample. Wilcoxon Signed Ranks tests between the scores for the first and last presentations of each predator showed that there was no significant effect of habituation for any measure and this applied to all of the model predators (*Z* scores ranged from: −0.031 to −1.512, *P* values ranged from 0.13 to 0.975). Two factors could have contributed to the absence of habituation: long intervals (minimum of 2 months) between presentations of the same predator and some variation (up to 20 m) between trials in the location at which the model was placed (Shalter, 1978).

As shown previously, discrimination between predators also extends to vocal behaviour (Kaplan et al., 2009). Magpies vocalise extensively during such agonistic encounters, as do other species, but in addition may also change their call type in accordance with predator type. The vocalisations elicited by eagles included a newly identified and very distinct call type, whereas harsh, noisy vocalizations were produced mostly when shown a Monitor Lizard (Kaplan et al., 2009), further showing that magpies’ perception of classes of predator (aerial versus terrestrial) guides their anti-predator strategy.

Discrimination between aerial predators was also evident in non-vocal responses of magpies. The Little Eagle elicited significantly more swoops than the Brown Goshawk. Although the magpies performed similar numbers of direct swoops at all raptor models, they performed significantly fewer looping swoops at the goshawk than at the other species of raptor.

Although the results demonstrate that the magpies discriminate between the eagles and the goshawk, there was no difference between the responses to the goshawk and the Wedge-tailed Eagle. Hence, these results do not support our hypothesis that mobbing intensity would correspond to level of threat determined by the prevalence of avian species within the diet of the predator species. Alternatively, the magpies may have been responding to the hunting techniques or flight abilities of the raptors. The goshawk is a fast, agile flier, certainly more so than the eagles, and thus warrants more caution during mobbing. In fact, fewer looping swoops were elicited by the goshawk than by the two eagle species. Since looping swoops are slow flights involving two passes over the predator, the strategy may be to avoid using these to mob an agile flier that could attack successfully during such close approaches. Kaplan and colleagues also found important additional differences in response to the Little Eagle versus the Wedge-tailed Eagle (Kaplan et al., 2009). Recruitment of conspecifics was significantly higher during presentations of the Little Eagle than during presentations of the Wedge-tailed Eagle. Such high recruitment of conspecifics occurred in regions where Little Eagles were particularly common (and more common than Wedge-tailed Eagles). Recruitment of conspecifics was observed in Wedge-tailed Eagles especially when the individual bird of prey was hidden from clear view (Kaplan, 2011) and there was a possibility that some magpies had failed to see it. Clearly, the aim of the attacks was to get predators to leave the magpies’ territory (Betts, Hadley & Doran, 2005) and the best chance of achieving such an outcome was by incessantly mobbing them.

## Conclusion

Overall, magpies show a sophisticated anti-predator repertoire and can readily adapt their behaviour depending on circumstances. The data present clear evidence that magpies vary their responses according to type of predator. They responded to the reptilian predators by either approaching them on the ground (the Monitor Lizard) or not at all (the snake). By contrast, magpies were rarely seen on the ground during presentations of any of the model raptors. Instead, they adopted an aerial strategy of swooping and adapted even their flight patterns in accordance with species.

Such discrimination has been found recently in Longfin Squid, *Loligo pealeii* (Straudinger, Hanlon & Juanes, 2011). By contrast, in most studies of mobbing, intensity of mobbing is usually described as the number of mobbing calls given (e.g. Owings & Loughry, 1985; Graw & Manser, 2007) or defined as the presence of any alarm calls (Edelaar & Wright, 2006). Mobbing intensity in the magpies cannot be accurately assessed by determining the number of mobbing calls alone, since this species uses non-vocal and vocal components (Kaplan et al., 2009) in one and the same response to a specific predator. Magpies may well encode information about the size of a predator, as has been found in black-capped chickadees (Templeton, Greene & Davis, 2005) but, perhaps more importantly, their strategies show that they understand the different kind of risks involved in responding to different avian and terrestrial predators. Distinction between the predators is not indicated by a change in the intensity of mobbing. Instead, the magpies selected strategies of mobbing specific to the species of predator. To our knowledge, this is the first time that such an arsenal of anti-predator defences, based on prior knowledge of predator behaviour, has been described in an avian species. The variation in the type of mobbing rather than varying mobbing intensity suggests a very complex and considered response to perceived risk.

## Additional Information and Declarations

### Field Study Permissions

The following information was supplied relating to ethical approvals (i.e. approving body and any reference numbers): License (S10361) issued by the Office of the Environment and Heritage, National Parks and Wildlife Service of NSW.

## References

[ref-1] Albrecht T, Klvaňa P (2004). Nest crypsis, reproductive value of a clutch and escape decisions in incubating female Mallards *Anas platyrhynchos*. Ethology.

[ref-2] Baker-Gabb DJ (1984). The feeding behaviour and ecology of seven species of raptor over winter in coastal Victoria. Australian Wildlife Research.

[ref-3] Betts MG, Hadley AS, Doran PJ (2005). Avian mobbing response is restricted by territory boundaries: experimental evidence from two species of forest warblers. Ethology.

[ref-4] Bravery JA (1970). The birds of Atherton Shire, Queensland. Emu.

[ref-5] Brooker MG, Ridpath MG (1980). The diet of the Wedge-tailed Eagle, *Aquila audax*, in Western Australia. Australian Wildlife Research.

[ref-6] Carrick R (1972). Population ecology of the Australian black-backed Magpie, royal penguin and silver gull. *Population ecology of migratory birds: a symposium*, U.S. Department of Interior Wildlife Research Report.

[ref-7] Coss RG, Ramakrishnan U (2000). Perceptual aspects of leopard recognition by wild bonnet macaques (*Macaca radiata*). Behaviour.

[ref-8] Creswell W (1993). Escape responses by redshanks, *Tringa totanus*, on attack by avian predators. Animal Behavior.

[ref-9] Curio E (1978). The adaptive significance of avian mobbing I. teleonomic hypotheses and predictions. Zeitschrift fur Tierpsychologie.

[ref-10] Curio E, Regelmann K, Zimmerman U (1984). The defence of first and second broods by Great Tit (*Parus major*) parents: a test of predictive socio-biology. Zeitschrift fur Tierpsychologie.

[ref-11] Debus SJS (1984). Biology of the little eagle on the northern tablelands of new South Wales. Emu.

[ref-12] de Azevedo C, Young RJ, Rodrigues M (2012). Failure of captive-born greater rheas (*Rhea americana*, Rheidae, Aves) to discriminate between predator and nonpredator models. Acta Ethologica.

[ref-13] Edelaar P, Wright J (2006). Potential prey make excellent ornithologists: adaptive, flexible responses towards avian predation threat by Arabian Babblers *Turdoides squamiceps* living at a migratory hotspot. Ibis.

[ref-14] Farabaugh SM, Brown ED, Hughes JM (1992). Cooperative territorial defence in the Australian Magpie, *Gymnorhina tibicen* (Passeriformes, Cracticidae), a group-living songbird. Ethology.

[ref-15] Graw B, Manser MB (2007). The function of mobbing in cooperative meerkats. Animal Behavior.

[ref-16] Griesser M (2008). Referential calls signal predator behaviour in a group-living bird species. Current Biology.

[ref-17] Hamerstrom F (1957). The influence of a hawk’s appetite on mobbing. Condor.

[ref-18] Helfman GS (1989). Threat-sensitive predator avoidance in damselfish- trumpetfish interactions. Behavioral Ecology and Sociobiology.

[ref-19] Kaplan G (2008). Australian Magpie: biology and behaviour of an unusual songbird.

[ref-20] Kaplan G, Johnson G, Koboroff A, Rogers LJ (2009). Alarm calls of the Australian Magpie (*Gymnorhina tibicen*): I. predators elicit complex vocal responses and mobbing behaviour. Open Ornithology Journal.

[ref-21] Kaplan G (2011). Pointing gesture in a bird- merely instrumental or a cognitively complex behaviour?. Current Zoologist.

[ref-22] King D, Green B (1999). Goannas: the biology of varanid lizards.

[ref-23] Kleindorfer S, Fessl B, Hoi H (2005). Avian nest defence behaviour: assessment in relation to predator distance and type, and nest height. Animal Behavior.

[ref-24] Knight RL, Temple SA (1986). Why does intensity of avian nest defense increase during the nesting cycle?. The Auk.

[ref-25] Koboroff A, Kaplan G (2006). Is learning involved in predator recognition? A preliminary study of the Australian Magpie *Gymnorhina tibicen*. Australian Field Ornithology.

[ref-26] Kruuk H (1976). The biological function of gulls’ attraction towards predators. Animal Behavior.

[ref-27] Leopold AS, Wolfe TO (1970). Food habits of nesting Wedge-tailed Eagles, *Aquila audax*, in South-eastern Australia. CSIRO Wildlife Research.

[ref-28] Marchant S, Higgins PJ (1993). Handbook of Australasian, New Zealand and Antartic birds. vol. 2 raptors to lapwings.

[ref-29] Martin P, Bateson P (1993). Measuring behaviour: an introductory guide.

[ref-30] Montgomerie RD, Weatherhead PJ (1988). Risk and rewards of nest defence by parent birds. The Quarterly Review of Biology.

[ref-31] Owings DH, Loughry WJ (1985). Variation in snake-elicited jump-yipping by black-tailed prarie dogs: ontogeny and snake-specificity. Zeitschrift fur Tierpsychologie.

[ref-32] Redondo T (1989). Avian nest defence: theoretical models and evidence. Behaviour.

[ref-33] Shalter MD (1978). Effect of spatial context on the mobbing reaction of pied flycatchers to a predator model. Animal Behavior.

[ref-34] Sharp A, Gibson L, Norton M, Ryan B, Marks A, Semeraro L (2002). The breeding season diet of the Wedge-tailed Eagle (*Aquila audax*) in Western New South Wales and the influence of the rabbit *Calicivirus* disease. Wildlife Research.

[ref-35] Shine R (1987). Ecological ramifications of prey size: food habits and reproductive biology of Australian Copperhead Snakes (*Austrelaps,* Elapidae). Journal of Herpetology.

[ref-36] Shine R (1989). Constraints, allometry and adaptation: food habits and reproductive biology of Australian Brownsnakes (*Pseudonaja*, Elapidae). Herpetologica.

[ref-37] Sordahl TA (2004). Field evidence of predator discrimination abilities in American Avocets and Black-necked Stilts. Journal of Field Ornithology.

[ref-38] Straudinger MD, Hanlon RT, Juanes F (2011). Primary and secondary defences of squid to cruising and ambush fish predators: variable tactics and their survival value. Animal Behavior.

[ref-39] Swaisgood RR, Owings DH, Rowe MP (1999). Conflict and assessment in a predator–prey system: ground squirrels versus rattlesnakes. Animal Behavior.

[ref-40] Swaisgood RR, Rowe MP, Owings DH (2003). Antipredator responses of California ground squirrels to rattlesnakes and rattling sounds: the roles of sex, reproductive parity, and offspring age in assessment and decision-making rules. Behavioral Ecology and Sociobiology.

[ref-41] Templeton CN, Greene E, Davis K (2005). Allometry of alarm calls: black-capped chickadees encode information about predator size. Science.

[ref-42] Weaver BW (1989). Diet of the lace monitor lizard (*Varanus varius*) in southeastern Australia. Australian Zoologist.

